# Meat bezoar due to inadequate mastication leading partial bowel obstruction: A case report

**DOI:** 10.1016/j.ijscr.2023.108775

**Published:** 2023-09-02

**Authors:** Tahmineh Tahouri, Ehsanollah Rahimi-Movaghar, Mohammad Reza Safaei Qomi, Sarvenaz Mehrabi

**Affiliations:** a Shahid Modarres Educational Hospital, Shahid Beheshti University of Medical Sciences, Tehran, Iran; b Department of Surgery, Farhikhtegan Hospital, Faculty of Medicine, Tehran Medical Science, Islamic Azad University, Tehran, Iran; c Department of Emergency Medicine, Arak University of Medical Sciences, Arak, Iran

**Keywords:** Bezoars, Intestinal obstruction, Abdominal pain, Case report

## Abstract

**Introduction:**

Gastrointestinal bezoars may occur in individuals with a normal gastrointestinal tract structure or as a result of gastrointestinal defects and disease. This rare condition initially presents with general abdominal pain, mimicking appendicitis in later stages. Recognizing this condition as a differential diagnosis in patients with abdominal pain can prevent delays in diagnosis and serious complications.

**Presentation of case:**

We report a rare case of a meat bezoar in a 52-year-old man presenting with acute and generalized abdominal pain at an emergency department.

**Discussion:**

We discuss gastrointestinal bezoars as a rare differential diagnosis of abdominal pain and acute abdomen in people with no pre-existing medical history, and the challenges that might be faced during diagnosis and treatment.

**Conclusion:**

Gastrointestinal bezoars are rare which makes the diagnosis challenging. Obtaining a complete history and a full examination with appropriate imaging could help the diagnosis.

## Highlights

• Bezoars are rare and usually show no symptoms that make them underdiagnosed in cases with abdominal pain.
• Bezoars can be symptomatic and cause serious complications with delay in diagnosis.
• Symptomatic bezoars may be misdiagnosed with appendicitis.
• Obtaining an accurate history of patients with abdominal pain is one of the most important parts of diagnosis for bezoars.
• Imaging will help diagnose bezoars in patients with general symptoms.

## 1 Introduction

Gastrointestinal bezoars comprise undigested or inedible materials found in various parts of the gastrointestinal tract [1]. While they may occur in individuals with normal gastrointestinal tract structure, certain factors increase the risk, including anatomical abnormalities, gastrointestinal motility issues, diabetes mellitus, excessive fiber intake, psychological conditions, and poor mastication [[2], [3], [4], [5]]. This report presents a rare case of meat bezoar in a 52-year-old man at Firuzabadi Educational Hospital in Tehran, Iran. This case is reported according to the Updating Consensus Surgical CAse REport (SCARE) 2020 guideline [6].

## 2 Case presentation

A 52-year-old man with no significant past medical history presented to the emergency department of Firuzabadi Educational Hospital in Tehran, Iran, with acute, generalized abdominal pain. The patient was not taking any medications, and there was no medical history suggesting an underlying condition related to chewing disorders. The patient reported no nausea, vomiting, or other gastrointestinal symptoms except for obstipation over the past three days. There was no tenderness, guarding, or rebound tenderness in the initial examination.

Laboratory assessment yielded normal results without any evidence of leukocytosis, raised inflammatory biomarkers, or abnormalities in urinary analysis. Standing abdominal and pelvic radiographs were normal showing no signs of pneumoperitoneum or air-fluid levels. Abdominal and pelvic ultrasound showed an inter-loop fluid collection in the right lower quadrant. Accordingly, the patient was admitted to the hospital.

During hospitalization, a serial examination of the abdomen was performed in terms of the presence of tenderness, rebound tenderness and guarding, all of which were normal. Serial rectal examination also revealed no abnormalities. However, after 24 h of hospitalization, the patient's abdominal pain intensified and localized to the right lower quadrant. Physical examination now revealed mild abdominal distension and right lower quadrant tenderness and rebound tenderness. Additionally, the patient had not tolerated oral intake for the past 12 h and had not defecated during hospitalization.

**Fig. 1:**
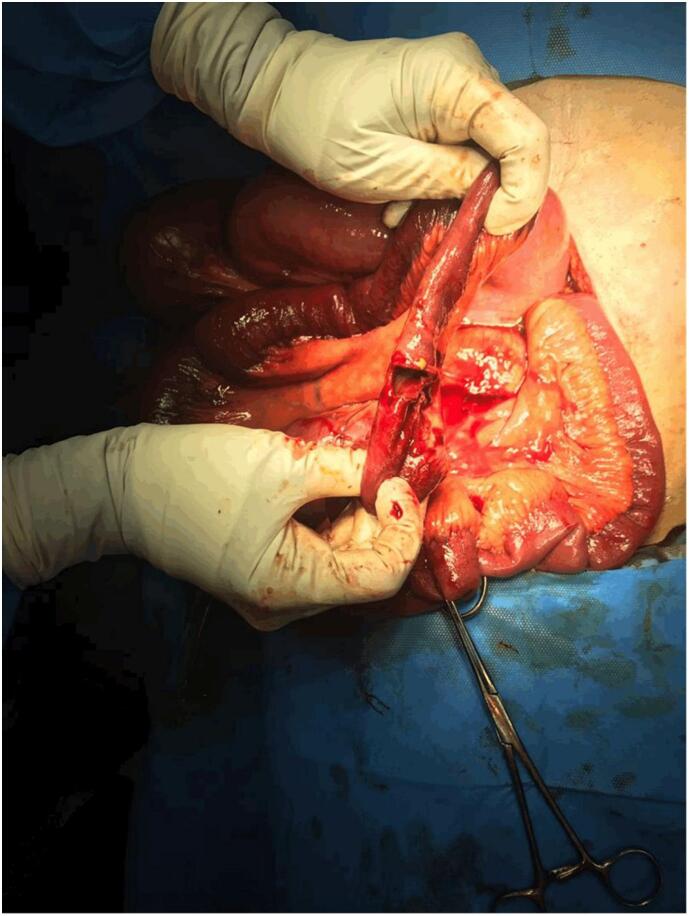
The obstruction region behind the ileocecal valve caused necrosis and sealed perforation.

**Fig. 2:**
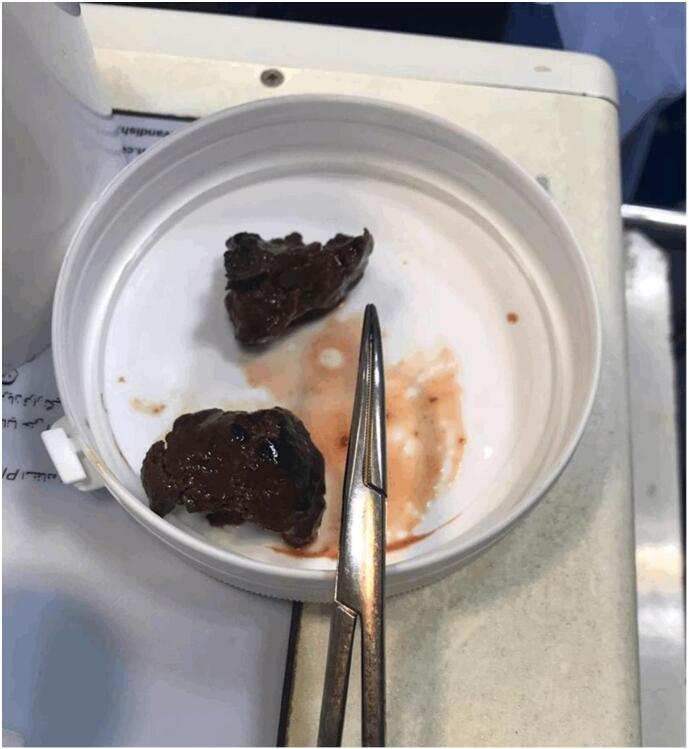
Two samples of Meat bezoars were extracted from the bowl.

Repeated abdomino-pelvic ultrasound showed dilated jejunum loops with no evidence of appendicitis. In our center, CT-san capabilities were unavailable. Due to severe abdominal pain and tenderness in the right lower quadrant, the patient's obstipation and oral intake intolerance, with suspicion of intestinal obstruction, the patient was immediately transferred to the surgery room and underwent exploratory laparotomy through a midline incision. During exploratory laparotomy, dilated small intestine loops with intraluminal masses in the ileum terminal were discovered. The ileum diameter before and after the obstruction site measured 5 cm and 1 cm, respectively. Additionally, necrosis, and sealed perforation were found 50 cm proximal to the ileo-ceal valve, where an Enterotomy was performed. The Enterotomy revealed large bezoar masses in front of the ileocecal valve, causing partial bowel obstruction (Fig. 1). A sample was resected and sent for pathological assessment (Fig. 2). The bezoar masses were successfully extracted, and since approximately 2 cm of the ileum showed necrosis, 50 cm from the ileocecal valve, resection of the necrotic segment and ileo-ileal anastomosis were performed. The enterotomy site was repaired in two layers using 2–0 Vicryl suture. To prevent any complications during postoperative recovery, a thorough examination of the small and large bowel was conducted before closing the abdomen.

Postoperative recovery proceeded smoothly. The patient was initially transferred to the intensive care unit and, on the second post-surgery day, to the surgery ward in stable condition. Due to mild ileus in the early post-operative period, a normal diet was not initiated until the fifth day after the surgery. On the fifth post-surgery day, the patient successfully tolerated a normal diet, and the abdominal skin was closed on the sixth post-surgery day. The patient was discharged the following day. The patient's medical history revealed that he had ingested Chenjeh Kebab, a kind of Persian roasted lamb pieces. According to the pathology report, inadequately masticated met pieces were identified as the etiology of bezoar formation. The patient underwent a follow-up two months later, and no clinical problems were observed.

The patient provided informed consent for reporting his condition and treatment approaches for educational and academic purposes.

## 3 Discussion

While very rare, cases of meat bezoars have been previously described. In a case presented by Henry et al. in 2016 [7], a 53-year-old man with a history of laparoscopic Roux-en-Y gastric bypass about 12 years prior was reported to have meat bezoar. Imaging revealed a diffusely dilated small bowel with a transition point in the distal ileum. Similar to the case in our study, Henry et al. suggested that inadequate mastication of chopped pork meat a few days before presentation, might have contributed to bezoar formation. it's important to note that bezoars can be formed not only though the ingestion of large amounts of indigestible or inadequately masticated foods, but also due to factors like motility disorders (secondary to previous gastric surgeries, hypothyroidism, and diabetes mellitus), reduced gastric acid secretion after vagotomy, and identified psychiatric disorders such as trichophagia, anxiety disorders, anorexia nervosa, and autism [[8], [9], [10]].

In our study, the patient initially presented with generalized abdominal pain. Despite normal laboratory and imaging reports, ultimately, the bezoar was diagnosed through exploratory laparotomy. Bezoars can consist of different components, such as indigestible vegetable or fruit fibers, hair, food particles, milk proteins, and even medications, resulting in varied presentations and side effects. To effectively manage gastrointestinal bezoars, especially, those related to inadequate food mastication, obtaining a detailed and accurate medical history is crucial. Bezoars may be asymptomatic or present with symptoms such as a palpable abdominal mass, gastric outlet obstruction, gastric pain, early satiety, weight loss, and even ulceration, perforation and hemorrhage. In some cases, the symptoms may be vogue or nonspecific, leading to a misdiagnosis or delayed diagnosis [11]. Since meat bezoar is very rare, we did not initially suspect it based on the patient's medical history. Furthermore, due to the patient's fatty abdomen, the bezoars were not palpable during abdominal examination. Therefore, arriving at a definitive diagnosis requires direct inquiries about the patients' diet, drug intake, and any material ingested in patients with unexplained abdominal pain [12].

Abdominal radiography has been found useful in suspecting bezoars in approximately half of the affected patients [13]. Ultrasound and computed tomography (CT) scans are reliable diagnostic tools for gastrointestinal bezoars [14], and endoscopy is also helpful for diagnosis [15]. Depending on the type and location of the bazoar, various medical and surgical methods are available for dissolution and/or retrieval. With the advancement of minimally invasive techniques, there have been recent reports of laparoscopy being utilized for both diagnosis and treatment in small bowel obstruction (SBO) cases. Laparoscopy not only enables a comprehensive evaluation of the cause, location, intestine viability, and obstruction severity, but it also guides the definitive treatment. Studies have demonstrated that laparoscopic treatment yields better postoperative outcomes compared to traditional open surgery. Moreover, the laparoscopic approach is particularly attractive as it reduces the risk of intra-abdominal adhesions formation following the procedure [16]. However, in the case presented here, since laparoscopy was not available at our center, surgical intervention was necessary due to the bazoar's dense content, acute abdomen presentation, and deterioration of clinical symptoms.

The meat bezoar in this case formed due to inadequate mastication without any pre-existing medical conditions. The delayed definitive diagnosis could have led to adverse outcomes. Nevertheless, the patient's clinical condition improved after the surgery, and he remained asymptomatic thereafter.

## 4 Limitations

This study has certain limitations that need to be acknowledged. Firstly, it is based on only one case, making it difficult to generalize the symptoms and findings to all other cases with similar presentations. Therefore, the findings should be interpreted with caution and considered more as a guiding case in its specific context.

Additionally, the study lacked an explicit scientific method for case presentation. To address this limitation, we followed the Updating Consensus Surgical CAse REport (SCARE) 2018 guideline, ensuring rigor in reporting and analysis [7].

## 5 Conclusion

Despite being a rare diagnosis in patients with abdominal pain, gastrointestinal bezoars should always be considered as a possible differential diagnoses due to their potential to cause bowel obstruction and serious complications. To effectively manage such cases, obtaining a complete medical history is essential, as it can guide the selection of appropriate approaches and diagnostic tests for these patients.

## Ethical approval

Ethical approval is not required for this study design in our institution.

## Funding

This research did not receive any specific grant from funding agencies in the public, commercial, or not-for-profit sectors.

## Author contribution

Tahmineh Tahouri: Study design and concept, supervising accuracy of data collection, data validation, and data analysis.

Ehsanollah Rahimi-Movaghar: Study design and concept, data collection and following-up, data analysis and interpretation, manuscript accuracy checking.

Mohammadreza Safaei Qomi: Data collection, writing and editing the manuscript.

Sarvenaz Mehrabi: Literature review, writing and editing the manuscript, manuscript accuracy checking. All authors confirmed the final manuscript and accepted its publication.

## Guarantor

Dr. Ehsanollah Rahimi-Movaghar

## Consent

The patient was informed about the intention to report his condition and the treatment approaches for educational and academic aims, and written informed consent was obtained for the publication of this case report.

## Conflict of interest statement

The authors declared no conflict of interest.
